# Psychological Reactance to Mobility Restrictions Due to the COVID-19 Pandemic: A Japanese Population Study

**DOI:** 10.3389/fpsyg.2021.655022

**Published:** 2021-06-11

**Authors:** Hiroyuki Sakai, Mariko Shimizu, Takayoshi Yoshimura, Eiji Hato

**Affiliations:** ^1^Toyota Central R&D Laboratories, Inc., Tokyo, Japan; ^2^Department of Civil Engineering, University of Tokyo, Tokyo, Japan

**Keywords:** reactance, motivation, restriction, self-restraint, COVID-19

## Abstract

In Japan, mobility restrictions were enforced by the government to abate the spread of COVID-19. The current study examined whether experiences of such mobility restrictions affected motivation for future going-out activities. To this end, we conducted a one-time online survey of 1,000 adults in Tokyo to measure going-out activities in four different time periods at once: before the spread of infection, during and after the emergency declaration, and after the end of the pandemic (future desire). In addition, to examine the impact of preferences for online services that make it easier to stay home, we measured the usage history of online services to obtain food during the mobility restriction period. Results indicated that desire for going-out activities after the end of the pandemic increased compared with those before the pandemic, particularly for leisure-related purposes. In addition, the use of online services to obtain food tended to suppress the increased desire for future going-out activities, although this effect was not significant. In conclusion, mobility restrictions resulted in motivational arousal for going-out activities after the end of the pandemic. Our findings indicate that psychological reactance plays a role in determining going-out activities in the future.

## 1. Introduction

During the first wave of the COVID-19 pandemic, individuals in many countries experienced mobility restrictions. In Japan, the government announced an emergency declaration (ED) requiring people in Tokyo to refrain from unnecessary going-out activities on April 7, 2020. In addition, various shops, restaurants, and leisure facilities were required to shorten their business hours or to temporarily close. Although the ED was lifted on May 25, the Ministry of Health, Labour, and Welfare urged Japanese citizens to adapt to a new lifestyle including self-restraint of going-out activities (https://www.mhlw.go.jp/content/10900000/000632485.pdf). Similar or even stricter mobility restrictions were imposed in other countries.

Such experiences of mobility restrictions could potentially suppress motivation for future going-out activities after the end of the pandemic. An increasing number of studies have examined the unprecedented growth of e-commerce with the spread of COVID-19 (Donthu and Gustafsson, 2020). In particular, a rapid increase in online food trade has been reported worldwide, including in China (Gao et al., 2020), Taiwan (Chang and Meyerhoefer, 2020), and Germany (Dannenberg et al., 2020). Because food purchasing is a critical everyday activity, if online shopping habits to obtain food become established, future going-out activities may be suppressed even after the pandemic ends. Decreased going-out activities have been reported to lead to reductions in physical activity levels (Ammar et al., 2020; Fukushima et al., 2020) and could therefore constitute a physical (Warburton et al., 2006) and mental (Biddle and Asare, 2011) health risk factor.

In contrast, psychological reactance theory (Brehm, 1966) predicts that mobility restrictions may facilitate motivation to perform going-out activities after the end of the pandemic. Psychological reactance is a state of unpleasant motivational arousal against threats to or loss of behavioural freedoms, with motivation directed toward recovering these freedoms (Brehm and Brehm, 1981). A number of studies have provided supportive empirical evidence for this theory. For instance, Miller et al. (2006) investigated risk factors for the initiation of smoking behaviours in adolescence, revealing that psychological reactance traits were a prominent predictor of potential smoking behaviour. Furthermore, Erceg-Hurn and Steed (2011) demonstrated that smoking cessation warning messages, contrary to their intention, elevated the craving for smoking. Psychological reactance to persuasive health communications has been repeatedly demonstrated in previous research (see Reynolds-Tylus, 2019 for review). Recently, Akhtar et al. (2020) delineated the psychological structure of consumers' psychological reactance toward the restoration of freedom in relation to offline shopping during the COVID-19 pandemic in a Chinese population. To our knowledge, however, there is no empirical evidence regarding psychological reactance in relation to restoring freedom of mobility.

In the current study, we thus carried out a one-time online survey investigating the desire for future going-out activities after the COVID-19 pandemic in a Japanese population. Specifically, we recruited 1,000 community-dwelling adults in Tokyo via the Internet and measured overall going-out activities and activities specific to leisure using the Life-Space Assessment (LSA) questionnaire (Baker et al., 2003) in four different time periods at once: before the spread of infection (baseline), during the ED, after the ED, and after the end of the pandemic (future desire). In addition, to examine the impact of preferences for online services that make it easier to stay home on the desire for going-out activities in future, we also collected data on the usage history of online services to obtain food during the ED.

## 2. Materials and Methods

### 2.1. Participants

**Figure 1:**
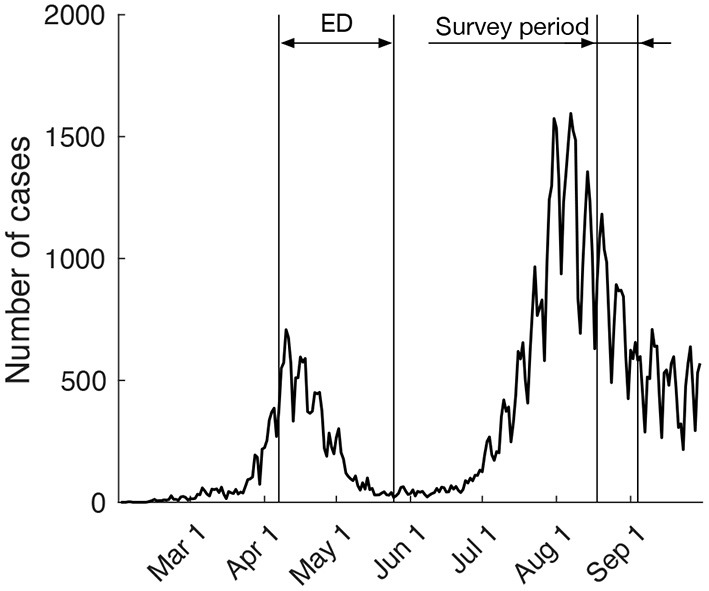
The number of daily COVID-19 cases in Japan. The Japanese government announced the ED requiring people in Tokyo to refrain from unnecessary going-out activities on April 7 and lifted it on May 25. Our online survey was carried out from August 18 to September 4. This survey period was ~3 months after the ED for the first wave of the COVID-19 pandemic in Japan and was conducted during the putative second wave. This plot is based on the data released by the Ministry of Health, Labour, and Welfare.

The present survey was carried out as part of a multipurpose survey that started on August 18, 2020 and ended on September 4, 2020 (Figure 1). It should be noted that the survey period was ~3 months after the ED for the first wave of the COVID-19 pandemic in Japan and took place during the putative second wave. In this survey, participants were recruited via a web-based survey site (Rakuten Insight, Inc., Tokyo, Japan). Among respondents, we excluded 160 males and 94 females because of obviously insincere responses (e.g., respondents for whom the elapsed time to complete was extremely short). We stopped recruitment after enrolling 1,000 community-dwelling adults who lived in the urban core in Tokyo (Chiyoda, Chuo, Minato, Shinjuku, Shibuya, Bunkyo, Taito, Toshima, Sumida, and Koto wards) with stratified sampling in terms of gender and age group (25–34, 35–44, 45–54, 55–64, and ≥ 65 years). Participants were offered financial compensation for completing the survey. The experimental protocols were approved by the ethical committee of Toyota Central R&D Laboratories, Inc.

### 2.2. Survey

We assessed going-out activities in four different time periods, before the spread of infection (in 2019), during the ED (April and May 2020), after the ED (June and July 2020), and after the end of the pandemic, using the LSA questionnaire. The original LSA questionnaire is a self-report measure for summarizing how far (five distance levels from room to outside of town) and how often (five frequency levels from never to daily) an individual travels in a specific time period (Baker et al., 2003). To examine going-out activities, we used items for three of the longer distance levels in the original questionnaire: in the neighbourhood (level 3), in town (level 4), and outside of town (level 5). In addition, although the original questionnaire assesses life space regardless of the purpose of movement, we used it to quantify going-out activities specific to leisure-related purposes, as well as activities for any purpose. For each time period and each purpose, an LSA score was calculated by adding the score for each distance level calculated as the product of the distance level (3–5) and the frequency level (0 = never, 1 = less than once a week, 2 = 1–3 times a week, 3 = 4–6 times a week, 4 = daily).

In this survey, we also collected data for self-reported usage history of online services to obtain food during the ED. Specifically, participants were asked to answer the following question: How did you eat during the ED? Please select up to three of the most applicable options from (1) You or someone you live with bought ingredients at supermarkets and cooked them at home. (2) You used online shops to buy ingredients. (3) You used food delivery services. (4) You ate out at restaurants. (5) You ate at someone else's home. Participants who selected both options (2) and (3) were classified into a group with a high preference for online services that made it easier to stay home; in contrast, participants who selected neither of options (2) or (3) were classified into a group with a low preference for online services that made it easier to stay home.

### 2.3. Data Analysis and Statistics

To compare going-out activities in different time periods, we performed a repeated-measures analysis of variance (ANOVA) on LSA scores separately for each purpose (overall and leisure). In this analysis, degrees of freedom were adjusted for sphericity using the Greenhouse-Geisser correction (Geisser and Greenhouse, 1958), and, if applicable, *post-hoc* multiple comparison tests were carried out using Shaffer's modified sequentially rejective Bonferroni procedure (Shaffer, 1986). In addition, we examined demographic effects on future desire for going-out activities by performing a two-way ANOVA with gender and age group on changes in LSA score in the future period relative to the baseline period. Furthermore, to examine the impact of preferences for online services on future desire for going-out activities, we compared changes in LSA scores in the future period relative to the baseline period between the low and high preference groups, using Welch's *t*-test. A significance threshold was set at *P* < 0.05 for all tests.

## 3. Results

In total, 1,000 participants (100 males and 100 females for each age group) completed the online questionnaire measuring going-out activities in the four different time periods, and preferences for online services to obtain food during the ED. According to the preference results, 602 participants (297 males and 305 females; mean age = 51.8 years, SD = 14.4) were classified into the low preference group, while 95 participants (40 males and 55 females; mean age = 45.3 years, SD = 12.1) were classified as the high preference group. The high preference group was significantly younger [*t*_(139.6)_ = 4.76, *P* < 0.001, Hedges' *g* = 0.46], compared with the low preference group.

**Figure 2:**
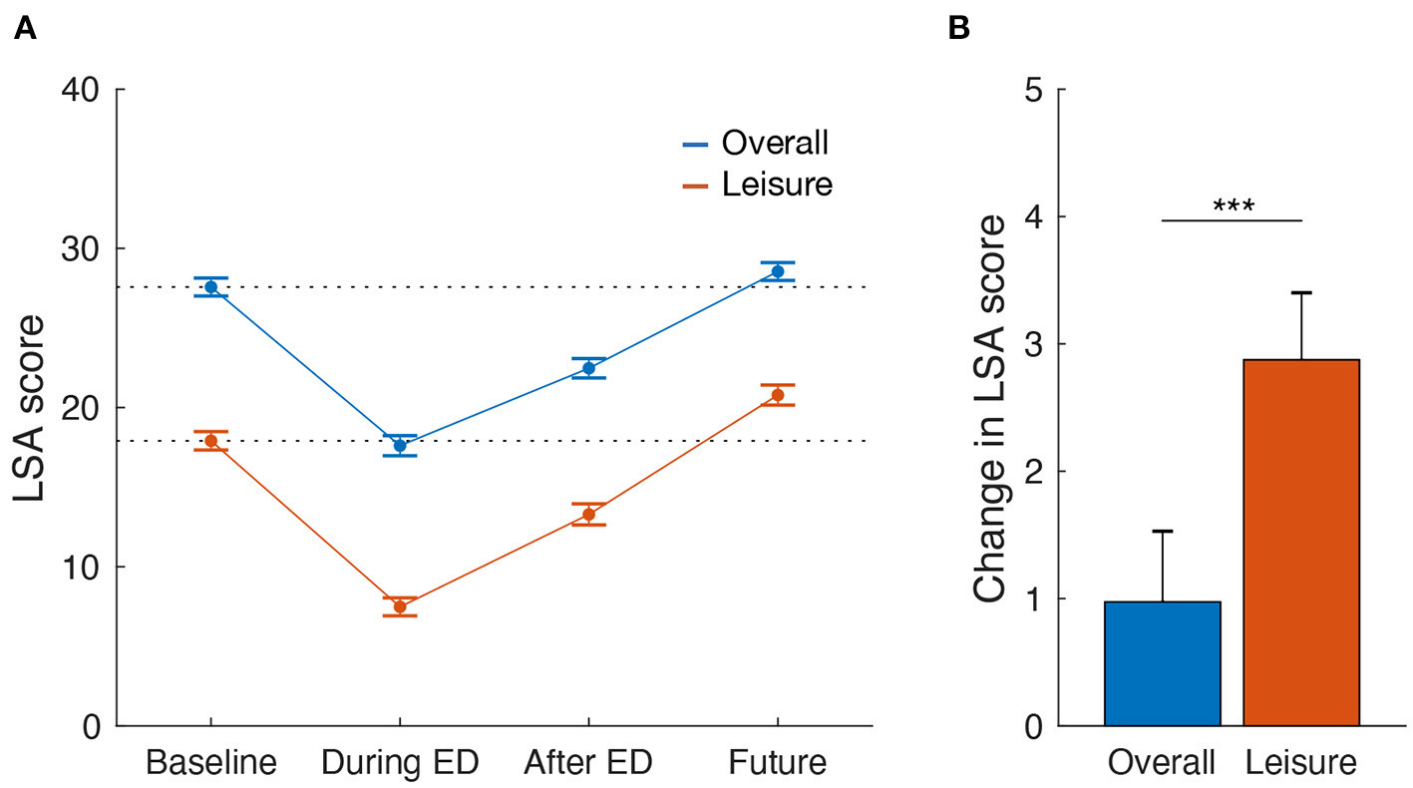
Impacts of mobility restriction due to the COVID-19 pandemic on going-out activities in Japan. Going-out activities were assessed with the LSA score in four different time periods **(A)**: before the spread of infection (Baseline), during the ED and after the ED to refrain from unnecessary going-out activities, and in the future after the end of the pandemic (Future). LSA score increases in the future relative to the baseline period were significantly larger for leisure purposes, compared with overall purposes **(B)**. Error bars represent 95% confidence intervals. Dotted lines indicate LSA scores in the baseline period. ***P < 0.001.

Regarding the overall going-out activities assessed with the LSA score, an ANOVA revealed a significant main effect of time period [$F_{\left(2.8,2969.7\right)}=954.92,P<0.001,\eta_{p}^{2}=0.49$] (Figure 2A). *Post-hoc* multiple comparison tests indicated that LSA scores during the ED significantly decreased relative to the baseline period [*t*_(999)_ = 36.07, *P* < 0.001, Cohen's *d* = 3.21] and subsequently, after the ED, LSA scores significantly recovered compared with those during the ED [*t*_(999)_ = 25.58, *P* < 0.001, *d* = 1.54], but did not reach the baseline level [*t*_(999)_ = 15.64, *P* < 0.001, *d* = 1.66]. Importantly, LSA scores in the future after the end of the pandemic were significantly higher compared with those in the baseline period [*t*_(999)_ = 10.72, *P* < 0.001, *d* = 0.32]. The time course of going-out activities specific to leisure purposes exhibited similar tendencies (Figure 2A). Thus, an ANOVA revealed a significant main effect of time period [$F_{\left(2.2,2211.1\right)}=668.55,P<0.001,\eta_{p}^{2}=0.40$]. *Post-hoc* multiple comparison tests indicated decreased LSA scores both during [*t*_(999)_ = 28.00, *P* < 0.001, *d* = 3.44] and after [*t*_(999)_ = 15.93, *P* < 0.001, *d* = 1.46] the ED, and increased LSA scores were observed in the future after the end of the pandemic [*t*_(999)_ = 3.44, *P* < 0.001, *d* = 0.92], relative to the baseline period. In addition, changes in LSA scores specific to leisure purposes in the future relative to the baseline period were significantly larger than those for overall going-out activities [*t*_(999)_ = 8.45, *P* < 0.001, *d* = 0.64] (Figure 2B).

**Figure 3:**
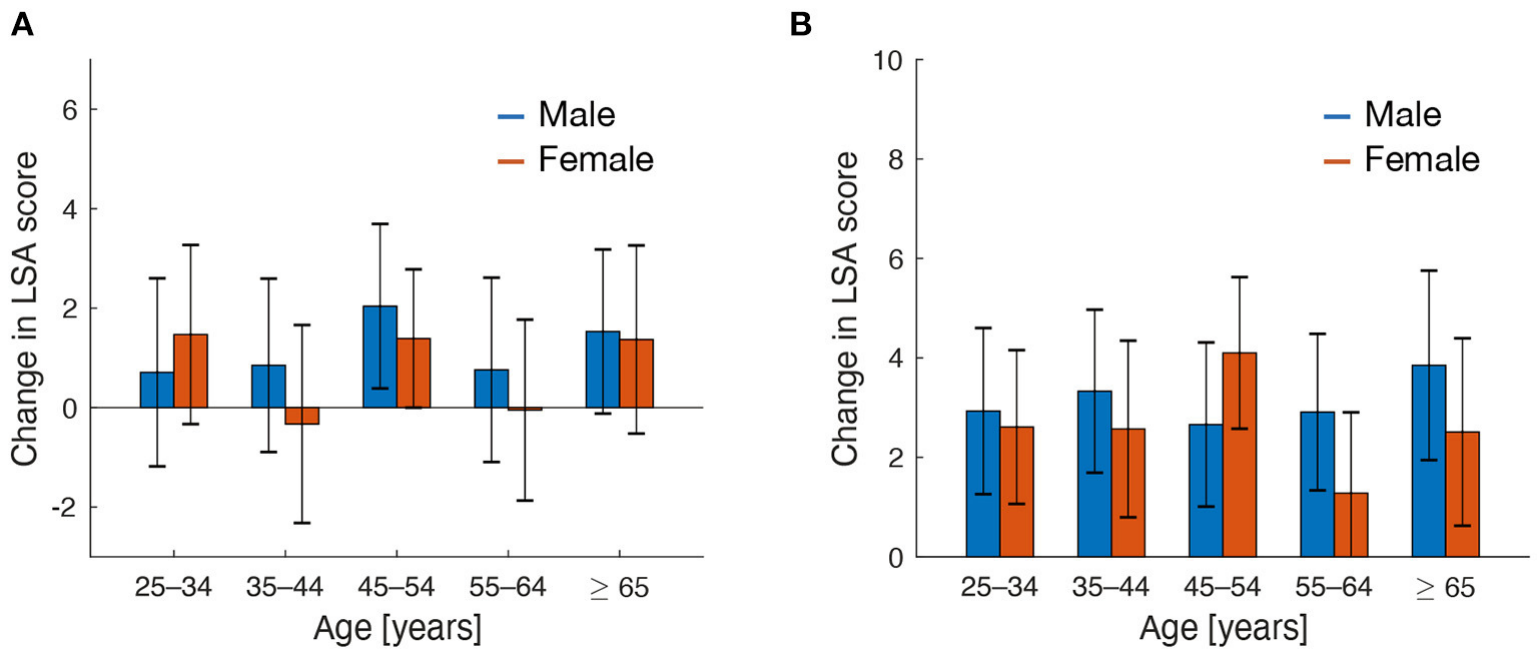
Going-out activities in the future after the end of the COVID-19 pandemic relative to those in the baseline period. Going-out activities assessed with the LSA score were compared in terms of age and gender. **(A,B)** Show changes in overall going-out activities and those specific to leisure purposes, respectively. Error bars represent 95% confidence intervals.

**Figure 4:**
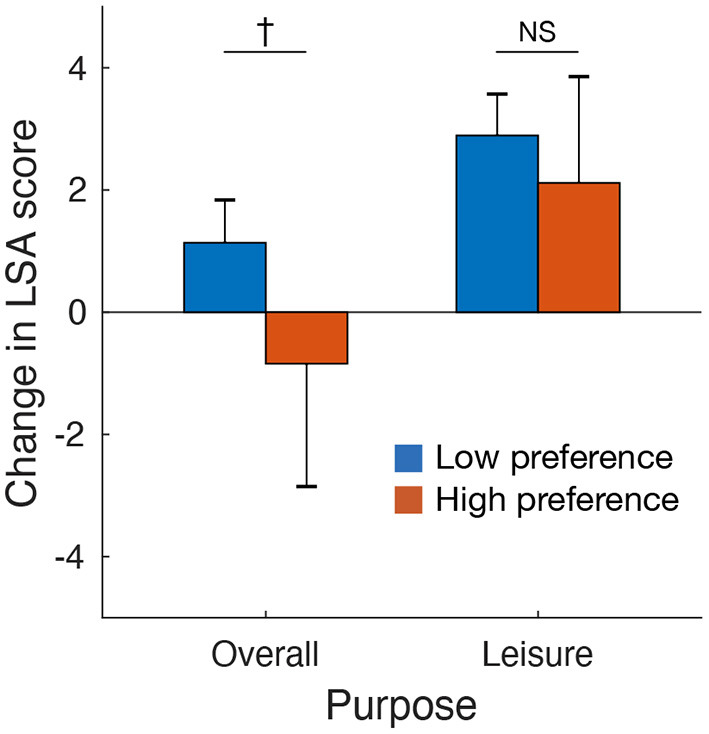
Impacts of preferences for online services to obtain food on desire for going-out activities in the future after the end of the COVID-19 pandemic. Participants were classified into high (*N* = 602) and low (*N* = 95) preference groups according to their usage history of online services to obtain food (e.g., food delivery) during the ED. Error bars represent 95% confidence intervals. ^†^*P* < 0.1; NS, not significant.

Moreover, we explored factors associated with reactance effects on going-out activities (i.e., increased LSA in the future relative to the baseline period). Regarding the effects of demographic factors on overall going-out activities, an ANOVA revealed no significant main effects of gender [$F_{\left(1,990\right)}=0.52,P=0.47,\eta_{p}^{2}=0.00$] or age group [$F_{\left(4,990\right)}=1.05,P=0.38,\eta_{p}^{2}=0.00$] and no significant interaction between gender and age group [$F_{\left(4,990\right)}=0.35,P=0.84,\eta_{p}^{2}=0.00$] (Figure 3A). For going-out activities for leisure-related purposes, the results revealed no significant main effects of gender [$F_{\left(1,990\right)}=0.95,P=0.33,\eta_{p}^{2}=0.00$] or age group [$F_{\left(4,990\right)}=0.68,P=0.61,\eta_{p}^{2}=0.00$] and no significant interaction between gender and age group [$F_{\left(4,990\right)}=1.01,P=0.40,\eta_{p}^{2}=0.00$] (Figure 3B). In contrast, preferences for online services to obtain food exhibited a weak but negative impact on reactant going-out activities in the future (Figure 4). The reactance effect on overall going-out activities was marginally smaller in the high compared with the low preference group [*t*_(118.5)_ = 1.84, *P* = 0.068, *g* = 0.22]. This decreased reactance effect was also observed in going-out activities for leisure-related purposes, but the difference was not significant [*t*_(124.9)_ = 0.82, *P* = 0.41, *g* = 0.087].

## 4. Discussion

In Japan, as in many other countries, governmental restrictions on going-out activities were enforced to abate the spread of COVID-19. In the current study, we examined whether experiences of mobility restrictions in a crisis situation affected individuals' motivation for future going-out activities in a Japanese population. The results revealed that desire for going-out activities after the end of the pandemic was increased compared with before the spread of COVID-19, particularly for leisure-related purposes. In addition, our data revealed that the use of online services to obtain food during the ED tended to suppress the increase in desire for future going-out activities, although this effect was not significant.

The current findings are in accord with psychological reactance theory (Brehm, 1966). During the ED, compared with the baseline period, going-out activities markedly decreased. This suggests that governmental restrictions worked as intended, and, at the same time, that behavioural freedom regarding going out was severely restricted during the ED. In contrast, the desire for going-out activities in the future after the end of the pandemic was increased compared with those during the ED, and, importantly, in the baseline period. These results indicate that experiences of mobility restriction stimulated the desire for going out in the future, in accord with the prediction of psychological reactance theory that loss of behavioural freedoms drives motivation to recover the freedoms. The current finding that increased motivation for going-out activities was more apparent for leisure-related purposes, which are determined by greater personal discretion, is also consistent with psychological reactance theory. Although psychological reactance to governmental restrictions of behavioural freedoms has previously been discussed in various contexts (Clee and Wicklund, 1980; Grandpre et al., 2003; Miller et al., 2006; Schade and Baum, 2007; Hornik et al., 2008; Shapiro et al., 2020), the current findings may constitute the first empirical evidence of psychological reactance to governmental mobility restriction in a crisis situation for public health.

In contrast to the desired going-out activities in the future after the end of the pandemic, going-out activities after the ED remained decreased compared with the baseline period. In accord with psychological reactance theory, it could be predicted that the level of going-out activities after the ED would immediately exceed that in the baseline period. However, it should be noted that the period after the ED (June and July, 2020) was during the putative second wave of the pandemic in Japan (Figure 1). Therefore, there was still a maintained focus of the Japanese media on the infection status of COVID-19. Considering these circumstances, our data can be interpreted as a result of self-restraint of going-out activities among people exposed to numerous daily reports about the pandemic. This interpretation supports the notion that reactance to restrictions does not always lead to direct restoration behaviours, but rather leads to restoration behaviours in more indirect ways, such as increasing an individual's preference for restricted choices (Brehm, 1966; Brehm and Brehm, 1981; Reynolds-Tylus, 2019). Thus, measuring respondents' desire for future going-out activities after the end of the pandemic was important for the purposes of the current research.

In the current study, no age or gender effect was observed in reactant behaviours to mobility restrictions. Because psychological reactance is a situation-specific state but also an individual trait (Brehm and Brehm, 1981), determinants of the reactant trait have been explored extensively (Seibel and Dowd, 2001; Buboltz et al., 2003; Seemann et al., 2005). However, demographic impacts on reactance are still under debate. Regarding gender effects, several studies reported that males had higher levels of reactance traits than females (Joubert, 1990; Seemann et al., 2004; Woller et al., 2007). In contrast, several other studies reported no significant gender differences (Brehm and Brehm, 1981; Hong, 1990; Hong et al., 1993). Research examining the effects of age on reactance is more scarce. Hong et al. (1993) found that reactance tended to decrease as age increased from 18 to 40 years. Woller et al. (2007) showed a U-shaped relationship between age and reactance, with older and younger adults exhibiting higher reactance than middle-aged adults.

The current findings do not completely exclude the opposite prediction that lifestyle changes due to mobility restrictions suppressed the desire for future going-out activities after the end of the pandemic. Although the difference was not significant, the current results suggested that the preference for online services to obtain food may have had a marginal negative impact on reactant going-out activities in the future. This tendency may become more prominent as the use of online services increases due to the prolonged COVID-19 pandemic. Because decreased going-out activities are a potential health risk factor (Ammar et al., 2020; Fukushima et al., 2020), promoting going-out activities after the end of the pandemic may be an ongoing public health challenge.

The current study involved several limitations that should be considered. First, the assessment of going-out activities using the LSA questionnaire may have been subject to response biases. In particular, because of social pressure to comply with government stay-at-home orders, participants may have underreported their going-out activities during the ED. If it occurred, this social desirability bias compromises our key assumption that freedom of mobility was severely restricted during the ED. However, there is evidence suggesting that going-out activities declined during the ED (Morita et al., 2020). Second, we examined psychological reactance to mobility restrictions by measuring the desire for future going-out activities. Further investigation will be needed to determine whether such indirect restoration behaviours result in an increase in actual going-out activities after the end of the pandemic. Although there is empirical evidence that self-reported life-space measures show good agreement with more objective measures derived from GPS data (Fillekes et al., 2019), this may not be applicable to the LSA score for future going-out activities because, for example, actual going-out activities can be limited by time and financial constraints. The use of GPS-derived life-space measures, instead of retrospective self-reports, would be useful to confirm and assess the robustness of the current findings. Third, we only examined a Japanese population. There is substantial evidence for cultural/ethnic differences in psychological reactance (Seemann et al., 2004; Woller et al., 2007; Ng et al., 2021). International comparison of psychological reactance against mobility restrictions due to the COVID-19 pandemic will be an interesting future research direction.

## 5. Conclusion

The current findings revealed that mobility restrictions due to the COVID-19 pandemic resulted in increased motivational arousal for going-out activities after the end of the pandemic in Japan. These findings highlight the role of psychological reactance in determining going-out activities in future, as well as indicating that the increasing spread of online services has the potential to mitigate such reactant going-out activities. A decrease in going-out activities would be expected to cause not only economic stagnation but also public health issues in relation to both physical and mental health. It will be important to continue examining going-out activities after as well as during the COVID-19 pandemic.

## Data Availability Statement

The raw data supporting the conclusions of this article will be made available by the authors, without undue reservation.

## Ethics Statement

The studies involving human participants were reviewed and approved by Ethical Committee of Toyota Central Research and Development Laboratories. Written informed consent for participation was not required for this study in accordance with the national legislation and the institutional requirements.

## Author Contributions

HS and MS participated in the design of the study, analysed all the research data, and drafted the manuscript. TY participated in the design of the study and reviewed the manuscript. EH reviewed the manuscript and supervise the study. All authors read and approved the final manuscript.

## Conflict of Interest

HS, MS, and TY were employed by Toyota Central R&D Laboratories. The remaining author declares that the research was conducted in the absence of any commercial or financial relationships that could be construed as a potential conflict of interest.
